# Release and recapture of silica nanoparticles from an optical trap in weightlessness

**DOI:** 10.1038/s41526-026-00596-y

**Published:** 2026-04-27

**Authors:** Govindarajan Prakash, Sven Herrmann, Ralf B. Bergmann, Christian Vogt

**Affiliations:** 1 Zentrum für angewandte Raumfahrttechnologie und Mikrogravitation (ZARM), Universität Bremen, Bremen, Germany; 2 BIAS—Bremer Institut für Angewandte Strahltechnik GmbH, Bremen, Germany; 3 Universität Bremen, Fachbereich 01: Physik/Elektrotechnik and MAPEX Center for Materials and Processes, Bremen, Germany

**Keywords:** Nanoscience and technology, Optics and photonics, Physics

## Abstract

Optically trapped silica nanoparticles are a promising tool for precise sensing of gravitational or inertial forces and fundamental physics, including tests of quantum mechanics at “large” mass scales. This field, called levitated optomechanics can greatly benefit from an application in weightlessness. In this paper, we demonstrate the feasibility of such setups in a microgravity environment for the first time. Our experiment is operated in the GraviTower Bremen that provides up to 2.5 s of free fall. System performance and first release-recapture experiments, where the particle is no longer trapped, are conducted in microgravity. This demonstration should also be seen in the wider context of preparing space missions on the topic of levitated optomechanics.

## Introduction

The unification of the theory of relativity and quantum mechanics remains one of the biggest challenges in today’s physics. For example the gravitational potential created by a massive particle in superposition is unknown^1^. In order to solve this puzzle scientists approach this interplay from two sides. On the one hand, the gravitational field of ever smaller source masses is investigated^2^, on the other, quantum mechanical behavior in ever larger mass systems is observed.

An established approach to prove quantum mechanical behavior is to observe interference of a system. Over the years this field was dominated by molecule interference from fullerenes in 1999^3^ to specifically tailored functionalized oligoporphyrins with masses of up to 25 kDa in 2019^4^. With growing complexity it became harder to launch these into an interferometer without destroying the fragile molecule structure, which led to the development of interferometry with metal clusters. This was successfully demonstrated recently, setting a new mass record of 172 kDa with sodium nanoparticles^5^. While these sources have a relatively high flux, the velocity spread and vacuum compatibility for UHV or below, might pose problems in the future.

Due to the possibility of highly-precise initial state preparation, a promising candidate to observe interference for even larger masses is levitated optomechanics^6,7^, where silica nanoparticles are trapped in an optical trap, formed by a focused laser beam. These systems are operated in vacuum chambers and can provide a high degree of isolation from environmental disturbances.

These properties are generally suitable for observing interference with relatively large masses, as interactions with the environment lead to decoherence and thus a smearing of the desired signal. In this context, the most important contributions to decoherence are collisions with gas molecules, as well as scattered, absorbed or emitted photons. In principle, it must be prevented that position information about the particle is available. While collisions can be prevented by an extremely good vacuum, an optical trap always provides a large number of photons that can be absorbed or scattered. It is therefore desirable to switch off the trap. Operation in zero gravity offers exactly this possibility, while the particle is still available for measurements for several seconds. These considerations led for example to the proposal of the MAQRO-mission, a dedicated satellite mission to test large mass particles for quantum mechanical behavior^8,9^, and a launched pathfinder mission to demonstrate particle loading in space^10^.

Another aspect to the presented work here is the possibility for measuring tiny forces with free-flying test particles. Hebestreit et al. demonstrated the measurement of the Earth’s gravitational acceleration with a free-falling nanoparticle^11^. The sensitivity of this measurement is limited by the available free-flying time, which could be extended by a movable trap^12^. Our approach in weightlessness in principle allows for extended free-fall times and increased force sensitivity.

Our experiment demonstrates for the first time the general feasibility of using levitated optomechanics in a weightlessness environment. In addition, we demonstrate first release and recapture experiments, paving the way to precise force measurements and “large” mass interference.

## Results and discussion

### Experimental setup

**Fig. 1:**
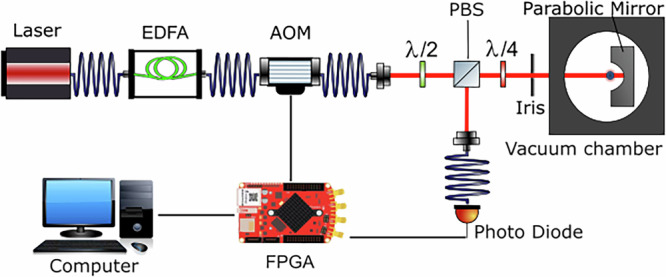
Schematic drawing. Experimental setup to optically trap silica nanoparticles in a vacuum. Detailed description in the text.

Figure 1 schematically shows our experimental design of an optical trap for transparent nanospheres in the focus of a laser while being operated in the weightlessness environment of the Drop Tower in Bremen.

Laser light at 1550 nm with a power of approximately 12 mW is generated by a fiber-coupled laser diode (Laser). The linearly polarized light passes an erbium-doped fiber amplifier (EDFA) providing approximately 500 mW to be fed into an acousto-optic modulator (AOM) for intensity control. From here, the light is guided as a free beam through a half-waveplate (*λ*/2) and a polarization-dependent beam splitter (PBS). Another quarter-waveplate (*λ*/4) converts the polarization from linear to circular. The collimated beam passes through an iris, necessary for our alignment procedure^13^, and hits a high numerical aperture (NA) parabolic mirror inside a vacuum chamber. The incoming light is focused and forms an optical trap for silica particles. The mirror is diamond-turned from aluminum with an NA higher than 0.9.

Backscattered light from the particle is collimated by the mirror and passes the waveplate again leaving a linearly polarized beam with perpendicular polarization to the incoming beam. Therefore, the beam splitter allows to separate incoming and outgoing beams. The scattered light is coupled into a single-mode fiber and guided to a single photodiode.

The photodiode provides an interferometric measurement between the scattered light, and a small fraction of the highly divergent field which passed the particle without scattering events^14^. Movement of the particle inside the optical trap translates to voltage differences on the photodetector, which are recorded on a digital oscilloscope running on a field-programmable gate array. The approach of a single photodiode was preferred over more sophisticated detection schemes, such as quadrant photo diodes^15^ or balanced photo detectors^16^ to align with the need for further compactification in future space missions. This approach allows for mode cleaning in a single mode optical fiber^17^, but forbids to assign possible offset shifts in the signal to single spatial directions (*x*,*y*,*z*), making it impractical for force measurements in the trapped state. Before recording, the photodiode signal passes a hardware highpass filter with a corner frequency of 1 kHz. When switching the laser off, this filter causes a sudden spike in the output signal, which rings down with a decay time of approximately 130 μs. A second, opposite spike appears when the laser is switched on again. The timespans without laser power presented in this paper are short compared to the ringdown time and the second peak is not visible in our measurements.

Particles with a diameter of (156 ± 5)nm are loaded into the trap by spraying them solved in ethanol with a nebulizer into the trapping region^18^.

The setup adapts current laboratory-based experiments for operation in microgravity. This includes miniaturized optic holders to minimize inertial force introduced by beam misalignment, all battery-powered devices and general compactification to meet the GraviTower requirements. An external turbo pump is used, which is disconnected for up to 3h during drop campaigns. In this time span the pressure inside the chamber rises linearly from approx. 2 × 10^−2^mbar to 3 × 10^−2^ mbar within the first 40 min. The pressure gauge has to be read visually from a display and can only be approximated for single experimental runs.

### Trap frequency measurements

The aim of our experiments is to demonstrate the feasibility of levitated optomechanical experiments in a microgravity environment. Therefore, trap frequency measurements at different laser powers were compared for flight and laboratory data and the particle’s movement in free flight is investigated.

Trap frequencies are resolved by a Fourier-transformation of the oscillatory interference signal recorded at the photodiode. For these measurements the incoming light was circularly polarized leaving all radial frequencies to be degenerate. No differences in trap frequencies are observed between the presence and absence of gravity. This is in accordance with numerical simulations of the particle’s movement in the trap, based on a second-order symplectic integrator^19^.

**Fig. 2:**
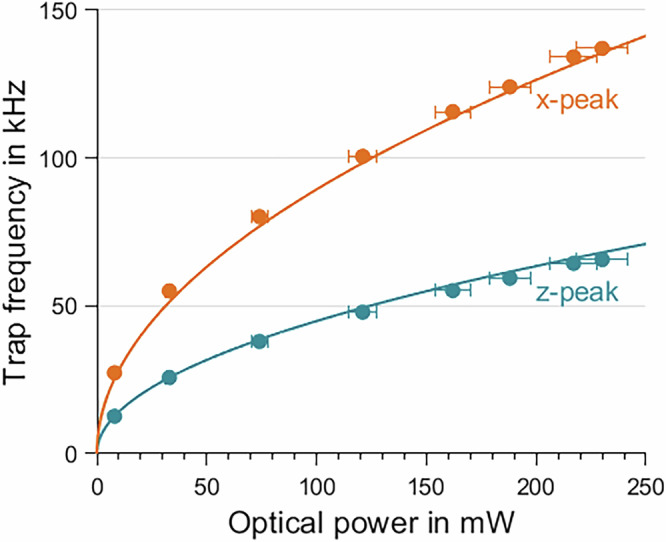
Trap frequencies. Measured in dependency of trapping power. Dots represent measurements while solid lines are fitted to Eq (1) leaving *ω*_0_ as free parameter.

Figure 2 shows resonance frequency of a particle in the trap in dependence on optical trapping power. The equations for radial (*ω*_*r*_) and axial trap frequency (*ω*_*ax*_) are

1 $${\omega }_{r}=\sqrt{\frac{4\alpha {P}_{0}}{m\pi c{\epsilon }_{0}{\omega }_{0}^{4}}}\,\,{\rm{and}}\,\,{\omega }_{ax}=\sqrt{\frac{2\alpha {\lambda }^{2}{P}_{0}}{m{\pi }^{3}c{\epsilon }_{0}{\omega }_{0}^{6}}}$$

where *α* and m are the real part of the polarizability of the particle and its mass, *c* is the speed of light, *ϵ*_0_ the electric constant and *P*_0_ the optical trapping power.

Fitting Eq. (1) to the data reveals a beam waist of *ω*_0_ = 0.7 μm meaning an effective numerical aperture of approximately 0.7. The discrepancy to the expected value seems to be a result of an improper alignment procedure^13^ and could not be fixed during the flight campaign. The iris just in front of the parabolic mirror was closed further than needed and therefore blocked some outer parts of the parabolic mirror.

By turning the *λ*/4 waveplate in front of the mirror, the trapping light becomes elliptically polarized, defining two orthogonal radial axes with different forces on the particle. The formerly degenerate radial trap frequencies split up into two different peaks in the spectrum. This is necessary, for example, to cool the particle’s motion in the future. For parametric cooling the trapping power is slightly modulated at twice the trapping frequency to reduce the particle’s motion, which only works for a known phase. This phase is not uniquely defined for degenerate radial trap frequencies^20^.

**Fig. 3:**
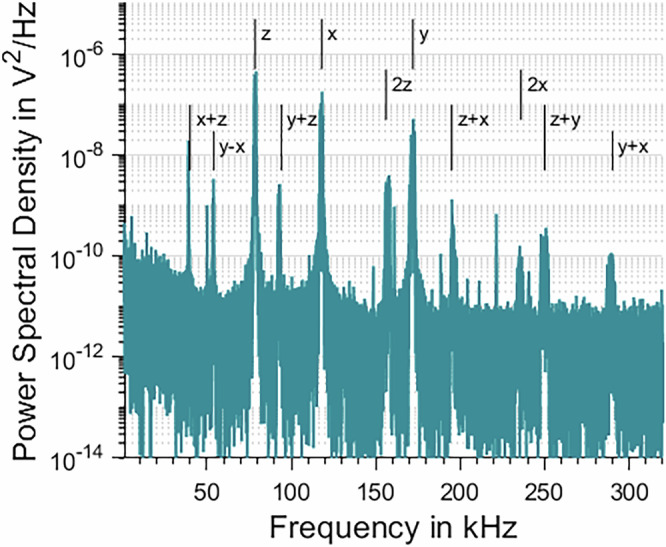
Power spectral density of the recorded interference signal for a particle oscillating in an anharmonic region of the optical trap. Fundamental frequencies belong to the three Cartesian axes and are labeled x, y, z accordingly. All other major contributions can be identified as higher harmonics or mixed terms of the fundamentals.

Figure 3 shows the resulting spectrum. The most dominant frequencies are fundamental trap oscillations in the three orthogonal directions defined by beam orientation and polarization, where z is aligned with the beam direction, x matches the semi-minor axis and y the semi-major axis of the elliptical polarization. Other peaks can be identified as higher harmonics or mixtures of the main trapping frequencies.

### Release—recapture experiments

**Fig. 4:**
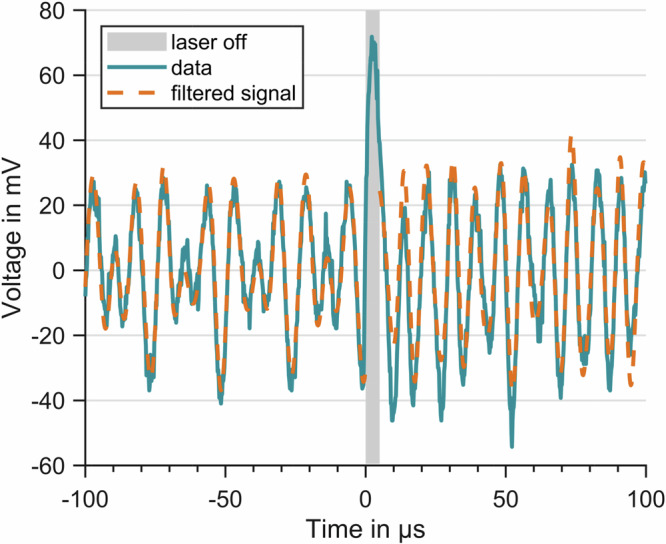
Photodiode signal. Recorded interference signal for a free flight measurement (solid, blue), where the trapping laser is switched off for 5 μs at *t* = 0 (shaded, gray). The signal is filtered (see chapter methods) to extract the movement in all three directions. Adding the filtered data up gives the dashed orange curve.

**Fig. 5:**
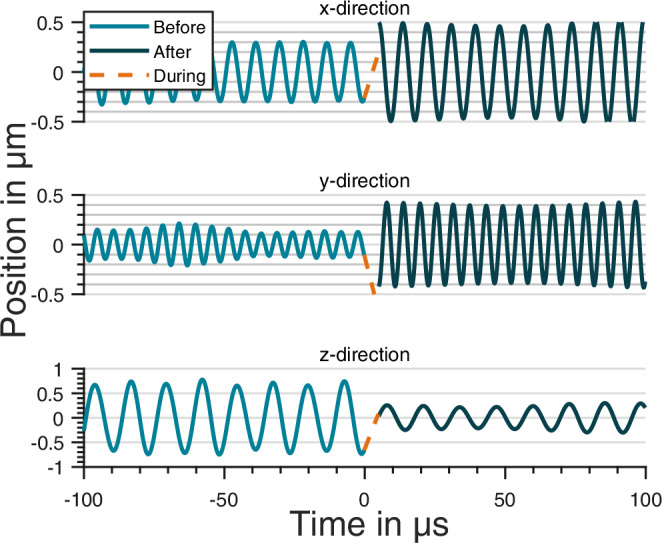
Free flight trajectory. Position data of the particle is separated for all three Cartesian axes. Data recorded before (blue) and after (black) the TOF are treated as different data sets. They are connected by the predicted free flight dynamics (orange dashed line) based on position and velocity at the switch-off time.

In our free flight experiments in microgravity the particle was released from the optical trap and recaptured, where the maximum available free flight time is limited by the kinetic energy of the particle. In the presented campaign, we were able to increase the laser off time from 1 μs to 10 μs and repeated the highest value six times before the particle was lost from the trap. An example of such an experimental run is shown in Figs. 4 and 5.

Figure 4 shows the recorded voltage over time as solid blue line. At *t* = 0 the laser is switched off. Since our trapping laser is used for position detection as well, we have no information about the particle during this time. The rising signal no longer represents the particle position but is the artifact caused by the high-pass filtering of the photodiode. From the trap frequency measurements, we expect a reduced laser power that is strong enough to allow for position determination to pose a significant force onto the particle.

After 5 μs the trapping laser is switched on and one can observe the particle’s oscillation again. Due to the free flying time, the particle has moved away from the center of the trap, resulting in a larger amplitude of oscillation. The resulting amplitude depends on the particle’s velocity, which in turn depends on the oscillation phase, at the time the trap is switched off.

In order to separate the different movements from each other, as seen in Fig. 5, we apply band pass filters (Butterworth of 10th order), centered around the main frequencies of the signal^21^. To test for consistency, all three filtered signals are added and compared to the recorded signal (orange dashed line in Fig. 4), giving normalized root mean square errors of 0.26 before the release and 0.36 after the recapture. A Fourier analysis shows the fundamental oscillations (*x*,*y*,*z*) as always present and dominant in our measurements.

A further movement into anharmonic trapping regions gives rise to mixed frequency components and higher harmonics. While higher harmonics can be fitted, added to the filtered signal and attributed to a single Cartesian coordinate, there is no straightforward procedure for mixed terms that we are aware of. Therefore, to cover movement in the strongly anharmonic regimes of the optical trap, it is more adequate to replace the single with a quadrant photodiode to record directional information directly. Our current system is only capable of reliable measurements in the small-amplitude (harmonic) regime. We observe that the filter-based separation of directional information breaks down once the mean voltage amplitude after recapture reaches approximately twice the mean value of the undisturbed trapped state over a time span of 3 s. In this regime, the oscillation amplitude becomes comparable to the beam waist and the particle explores strongly anharmonic regions of the optical potential. From data taken on ground we observe one third of experimental runs in the 5–6 μs free flight range to be usable with this approach. In agreement with these findings, we can investigate three out of nine experimental runs from the drop tower campaign. 9 μs and longer attempts could not be separated, neither on ground nor in the drop tower. This is caused by a longer distance covered by the particle in the longer free time. It is worth mentioning that, for nonlinear signals, we cannot technically distinguish whether they stem from an anharmonic trapping region or from nonlinearity in our detection system.

The interference signal was converted to position information, taking advantage of the known kinetic energy of the particle at room temperature^22^. Taking the mass and fitting parameter uncertainty into account the conversion factor is determined with an uncertainty of ±15% in z- and y- and ± 20% in x direction. The calculated position and velocity data can be used to predict the particle’s motion while the trap is switched off. In the free-flying times presented here, we expect residual forces acting on the particle (e.g., electrostatic forces) to be below our measurement sensitivity and therefore assume linear movement from the position and with the velocity in the exact moment the laser is switched off. Switching dynamics in the laser are ignored.

Figure 5 displays the particle’s position for all three Cartesian axes over time from the same data set seen in Fig. 4. While data before (blue) and after (black) the free flight are treated as separate measurements, the dashed orange line is the predicted free flight. To quantify the quality of our measurements, we post-corrected the phase of the oscillation before the switch-off to match the particle position after retrap with the first measured position after recapture. For ground data including gravity, the phases in all three dimensions have to be corrected by (0.25 ± 0.33), (−0.07 ± 0.32) rad and (−0.23 ± 0.56) rad for x,y and z, respectively. Our drop tower data, including the displayed, fall within this range. Notice, that the release of a particle does not necessarily lead to an increase in energy, but with the right timing can also lead to a center of mass cooling effect as observed here in z direction.

We demonstrated the first levitated optomechanics experiment in microgravity. This approach is expected to become important to provide undisturbed free evolution of nanoparticles as quantum mechanical wave packets. Such an observation would be useful for fundamental physics questions as well as a new kind of force sensors based on matter-wave interferometry. Free flights in microgravity of up to 10 μs were observed. This time is limited by the particle’s velocity. We demonstrated a single photo detector to be sufficient to describe the particle’s motion in all three translational degrees of freedom, if the movement is restricted to the harmonic and slightly anharmonic region of the trap. These movements can well be described and are consistent with expected free flight trajectories.

In the next step, the setup will be upgraded with an algorithm to provide parametric feedback cooling, which was demonstrated to reach temperatures of a few mK, translating to residual velocities of only hundreds of $\frac{\mu {\rm{m}}}{{\rm{s}}}$^14^. Since 100$\frac{\mu {\rm{m}}}{{\rm{s}}}$. 1 ms = 200 nm < *ω*_*o*_, such improvements would allow to extend the free flying time to the ms range in our setup.

## Methods

The interferometric signal between scattered light from the particle and reflected light from the mirror is recorded with a single photodiode connected to an oscilloscope (Picoscope, PS 4424A) with a sampling rate of 5$\frac{{\rm{MS}}}{{\rm{s}}}$. The information is further processed in MATLAB. A Fourier transformation on data of up to 1 s is used to extract the main frequency components in the signal and determine the fundamental frequencies in all three directions.

The original datasets with a duration of 10s are cropped to a total of 200 μs around the trapping laser switch-off time. The exact switch off is calibrated and post-corrected in time by the strong signal rise observed for every experimental run. For data before the trapping laser switch off, three different 10th-order Butterworth filters with half-power frequency values of 5kHz to both sides of the resonance are sequentially applied. The results are corrected for filter-introduced phase shifts by comparing the output with the original data based on correlation analysis. Since these filters always start at 0 amplitude and need some time to represent the recorded data appropriately, the data set from retrapping the particle was inverted in time before the same set of filters was applied.

The underlying dataset measured on ground to determine the average percentage of usable experimental runs consists of 64 experimental runs including six repetitions for 5 μs and 21 repetitions for 6 μs of free flight with 9 usable measurements (33%) in this range. A subset of 16 measurements (ranging 5–8 μs) from the entire dataset, which met the criteria for filter application was used for the determination of phase correction and therefore to estimate the quality of the method.

The normalized root mean square error for measuring the quality of our filtered results is defined here as:

2 $$NRMS=\frac{\sqrt{\langle {({x}_{data}-{x}_{fit})}^{2}\rangle }}{\sqrt{\langle {x}_{data}^{2}\rangle }}$$

### The GraviTower Bremen Pro

The GraviTower Bremen Prototype is a second-generation drop tower with a height of 16 m. With an initial acceleration of up to 5 g it provides up to 2.5 s of weightlessness on a parabolic vertical trajectory^23^. In contrast to first-generation drop towers, the new generation is actively driven to compensate for air drag. This eliminates the need for a vacuum environment and greatly reduces the experimental cycle by a factor of approximately 50. A sledge, running on Polytetrafluoroethylene skids, is driven by ropes connected to a hydraulic wrench. Residual vibrations are further reduced by a free-flying system inside the sledge decoupled by several air bearings. The initial acceleration can be chosen between 1.5 g and 5 g. For the work in this paper, the acceleration was fixed to 1.5 g, providing approximately 1.5 s of free flight. While this value is long enough for the performed experiments, the system was also capable of performing equally well with accelerations of up to 3.5 g, higher values were not tested.

The GraviTower sets certain requirements for our experiment. The maximum volume of 0.85 m^3^ and the maximum weight of 500 kg can easily be met. The system must be mounted on Aluminum/Plywood platforms with diameters of approximately 70 cm. Different platforms are connected via four aluminum stringers with a length of 1.34 m^24^. Furthermore, the setup must have autonomous power supply and run sequences with a single starting trigger.

## Data Availability

The data supporting the conclusions of this article are publicly available from 10.5281/zenodo.19076756.
